# Assembly, annotation, and comparison of *Macrophomina phaseolina* isolates from strawberry and other hosts

**DOI:** 10.1186/s12864-019-6168-1

**Published:** 2019-11-04

**Authors:** Alyssa K. Burkhardt, Kevin L. Childs, Jie Wang, Marina L. Ramon, Frank N. Martin

**Affiliations:** 1 Crop Improvement and Protection Research Unit, USDA-ARS, Salinas, California, USA; 2 Department of Plant Biology and Center for Genomics-Enabled Plant Science, Michigan State University, East Lansing, MI USA

**Keywords:** *Macrophomina phaseolina*, Genome assembly, Genome annotation, Strawberry, Host preference, Comparative genomics

## Abstract

**Background:**

*Macrophomina phaseolina* is a fungal plant pathogen with a broad host range, but one genotype was shown to exhibit host preference/specificity on strawberry. This pathogen lacked a high-quality genome assembly and annotation, and little was known about genomic differences among isolates from different hosts.

**Results:**

We used PacBio sequencing and Hi-C scaffolding to provide nearly complete genome assemblies for *M. phaseolina* isolates representing the strawberry-specific genotype and another genotype recovered from alfalfa. The strawberry isolate had 59 contigs/scaffolds with an N50 of 4.3 Mb. The isolate from alfalfa had an N50 of 5.0 Mb and 14 nuclear contigs with half including telomeres. Both genomes were annotated with MAKER using transcript evidence generated in this study with over 13,000 protein-coding genes predicted. Unique groups of genes for each isolate were identified when compared to closely related fungal species. Structural comparisons between the isolates reveal large-scale rearrangements including chromosomal inversions and translocations. To include isolates representing a range of pathogen genotypes, an additional 30 isolates were sequenced with Illumina, assembled, and compared to the strawberry genotype assembly. Within the limits of comparing Illumina and PacBio assemblies, no conserved structural rearrangements were identified among the isolates from the strawberry genotype compared to those from other hosts, but some candidate genes were identified that were largely present in isolates of the strawberry genotype and absent in other genotypes.

**Conclusions:**

High-quality reference genomes of *M. phaseolina* have allowed for the identification of structural changes associated with a genotype that has a host preference toward strawberry and will enable future comparative genomics studies. Having more complete assemblies allows for structural rearrangements to be more fully assessed and ensures a greater representation of all the genes. Work with Illumina data from additional isolates suggests that some genes are predominately present in isolates of the strawberry genotype, but additional work is needed to confirm the role of these genes in pathogenesis. Additional work is also needed to complete the scaffolding of smaller contigs identified in the strawberry genotype assembly and to determine if unique genes in the strawberry genotype play a role in pathogenicity.

## Background

*Macrophomina phaseolina* is a haploid, clonally reproducing ascomycete fungus that causes damping off, stem rot, and charcoal rot on a wide range of over 500 host species including soybean, corn, wheat, and strawberry [1–3]. This pathogen is soilborne and can survive multiple growing seasons by forming resting structures called microsclerotia, which are melaninized structures formed from 50 to 200 cells [3]. Some studies have shown that the survival and symptom severity caused by *M. phaseolina* increases with warmer soil temperatures, ranging from 28 to 35 °C [4, 5]. Typically, *M. phaseolina* has been thought to be a broad host range pathogen, with the same isolate being able to infect multiple plant species and thus posing a risk to growers planting a variety of crops in a field with a history of *M. phaseolina* [1, 6]*.* However, recent studies suggest that *M. phaseolina* may exhibit some degree of host preference and that each isolate may not pose an equal risk to all crops [7]. Specifically, our recent work studying *M. phaseolina* collected from strawberry and other hosts around California support the hypothesis that some isolates of *M. phaseolina* exhibit a strong host preference toward strawberry [8].

Recently, an increased incidence of *M. phaseolina* in strawberry fields has been observed as growers shift away from using methyl bromide as a preplant fumigant as required by changes in pesticide regulations [8–10]. As a result, the 2.3-billon dollar, 15,000-ha strawberry industry in California [11] is under increasing threat from this pathogen that causes crown rot and death of the plant. Interestingly, this pathogen is found in cooler, coastal strawberry growing regions of California despite its documented preferences for warmer climates [4, 5]. A large population study to understand potential regional and host differences of *M. phaseolina* from strawberry and other hosts growing mainly in California was conducted using SSR markers [12]. The results from an initial study using 65 SSR markers and 15 California isolates show that the majority of isolates recovered from strawberry cluster within a distinct clade [8]. A larger, unpublished study using 24 SSR markers and over 460 *M. phaseolina* isolates from California and other parts of the world support the grouping of most strawberry isolates into a single genotype (Marina Ramon and Frank Martin, personal communication). Understanding the genetics behind this single genotype grouping might lead to a better understanding of why some isolates of *M. phaseolina* exhibit a strong host preference for strawberry despite *M. phaseolina* being typically understood as a broad host range pathogen.

To begin to answer genomic questions, a high-quality, complete reference genome is needed to which other isolates can be compared. Prior to this study, the only published draft genome of *M. phaseolina* was sequenced from an isolate recovered from jute [1]. This isolate was sequenced using older technology, including Illumina and 454, resulting in a 48.9 Mb assembly consisting of 1506 contigs with an N50 of 151 kb [1]. The goal of the current study was not only to sequence isolates of *M. phaseolina* representing the main strawberry genotype and other genotypes, but also to use modern advances in sequencing technology, including long read PacBio sequencing, to provide a higher quality assembly with fewer fragments and more complete contigs. In recent years, the use of long read PacBio sequencing technology has lived up to the promise of delivering more complete genomes and has successfully been used to de novo assemble or dramatically improve the assembly of several plant-pathogenic fungi including *Verticillium dahliae* [13]*, Botrytis cinerea* [14]*, Fusarium oxysporum* [15]*, Magnaporthe oryzae* [16], *Sclerotinia sclerotiorum* [17], and *Colletotrichum higginsianum* [18]. In fact, Faino et al. 2015 found that for *V. dahliae* an assembly based on PacBio data alone was of better quality than an assembly that was created from a hybrid of PacBio and Illumina data.

To achieve the goal of producing a very high-quality reference genome for *M. phaseolina*, an isolate recovered from strawberry in Santa Barbara county in 2011, 11–12, was selected to provide the DNA for the reference PacBio assembly because it was representative of the strawberry genotype and exhibited a host preference for strawberry [8]. In addition, an updated structural annotation of the genome was completed in this study using ten separate RNA-Seq libraries of the 11–12 fungal tissue grown in different conditions to generate strong transcript evidence.

Further improvement of the PacBio-based genome assembly was done using Illumina-based mate pair and paired-end sequencing in order to correct any sequencing errors and further improve the quality of the final assembly [19]. In addition, Dovetail Genomics (Santa Cruz, CA) Hi-C technology was used to join contigs into scaffolds, break any contigs that were erroneously joined in the initial assembly, and fill in gaps, thereby improving the long-range scaffolds of the assembly [20, 21]. Because assemblies made with these new technologies provide significant advantages when investigating genome structure but may have sequence and structural bias when compared to Illumina-based assemblies, the genome of a second isolate of a different genotype named Al-1, which was recovered from alfalfa in California in 2013, was also assembled as described above for the 11–12 isolate. Together, the data from these two isolates were assembled into high-quality genomes which were used to investigate how structural changes in the genome may provide the basis for potential mechanisms of genome evolution in this asexual fungus that could lead to host preference. Overall, the goals of the work in this manuscript were to 1) provide a complete high-quality genome assembly for *Macrophomina phaseolina,* 2) provide an updated annotation for *M. phaseolina* and 3) investigate the genomic changes that may have contributed to strawberry host preference of some isolates of *M. phaseolina*.

## Results

### Genome assembly

In the process of generating the final assemblies for isolates 11–12 and Al-1, several methods and iterations of assembly were used to generate the highest quality assembly. Before the FALCON-based assemblies were selected as the final assembly, the HGAP assembly pipeline was used to generate an assembly for the 11–12 isolate with the PacBio data, but it had a lower N50 (3.3 Mb) than the FALCON assembly (N50 = 4.3 Mb) and did not run successfully with input data for isolate Al-1. The Genome Finishing Module of the CLC Genomics Workbench (Qiagen, Redwood City, CA) was also used to assemble both genomes using the PacBio data, but in both cases resulted in an assembly with a shorter total length (44 Mb) and over 120 contigs in each assembly. After the FALCON-based assemblies were selected as the best PacBio-based assemblies, a polishing run with PILON using paired-end and mate-pair Illumina reads greatly improved the base calling, with 113,895 and 73,557 SNP and indel changes made after the first PILON run for the Al-1 and the 11–12 assemblies, respectively. A second PILON polishing step was done and yielded a higher percentage of Illumina reads mapping. In this step, 3984 and 3784 additional SNP changes were made by PILON in the Al-1 and the 11–12 assemblies, respectively, resulting in the final highest quality, polished FALCON-based assembly.

The final PILON assemblies were further improved using Dovetail HiRise with PacBio gapfilling to combine some contigs into scaffolds. For 11–12, the input assembly of 102 contigs was broken once and joined 19 times, resulting in 84 total contigs/scaffolds. The joins made by the initial Dovetail HiRise scaffolding were made by adding 100 Ns at the junction, but 6 of these joins were subsequently filled in with the PacBio data from the initial sequencing, leaving 13 total gaps filled with 25–100 Ns. For Al-1, the input assembly of 27 contigs was not broken, but a single join was made with Dovetail HiRise resulting in 26 final contigs. The gap created by this contig joining was eliminated by the PacBio gapfill resulting in a final assembly of isolate Al-1 with no gaps and no Ns. To investigate the identity of the smaller (< 100 kb) contigs in each assembly, the assemblies were aligned to themselves using a BLAST-based tool in CLC Genomics Workbench, and contigs that were 99.5% contained within another contig and had greater than 99.5% identity to that contig were considered assembly errors and were eliminated along with contigs less than 1000 bp. After the Dovetail and CLC analyses of the genome, the final contig/scaffold counts were 60 and 18 for the 11–12 and Al-1 genomes, respectively. All of the contig/scaffolds and their lengths can be found in Additional file 1: Table S1. For ease of reference, the large segments of DNA in the final assemblies, which are largely gap-free, have all been named as “Contig #” with the Contig 1 being the longest contig for each assembly and Contig *n* being the shortest contig for each assembly with *n* representing the total number of contigs. The genome statistics for the final assemblies can be found in Table 1.

**Table 1:** Genome statistics for *Macrophomina phaseolina* isolates from strawberry that represent the strawberry genotype (11–12) and isolates recovered from diseased alfalfa (Al-1) and jute that represent other genotypes

Isolate	Genome Size (Mb)	# of Contigs/Scaffolds	N50 Mb	L75	Long Contig Mb	% 11–12 reads mapped	% Al-1 reads mapped	BUSCO euk	BUSCO fungi	# of genes
11–12	51.3	60^a^	4.3	8	6.8	96.4%	93.7%	98.3%	99.3%	14,103
Al-1	49.8	18^b^	5.0	8	6.8	85.0%	98.1%	98.3%	99.0%	13,443
Jute^c^	48.9	1506	0.15	205	1.1	84.9%	93.5%	98.3%	98.3%	14,249

^a^One contig represents the mitochondrial genome

^b^Four contigs represent the mitochondrial genome

^c^Islam et al. [1]

High-quality, nearly complete assemblies were generated for both the *M. phaseolina* isolate from strawberry, 11–12, and the isolate from alfalfa, Al-1 (Table 1). Both isolates had very high N50 scores, at 4.3 Mb and 5.0 Mb for the 11–12 and Al-1 isolate, respectively. The sequence coverage of the *M. phaseolina* genomes from strawberry and alfalfa was 150x for the PacBio reads and over 200x for the Illumina reads. The distribution of the lengths of the main contigs for 11–12 and Al-1 indicate that there are 13 large contigs that are all 870 kb - 6.8 Mb. The smaller 47 contigs of 11–12 range from 4726 to 99,276 bp with Contig 15 (94,974 bp) being the mitochondrial genome. The 5 smaller contigs of Al-1 range from 2215 to 49,130 bp with Contigs 14, 16, 17, and 18 representing the mitochondrial genome. No telomeres were detected in the 11–12 assembly, but 7 out of 14 nuclear contigs of the Al-1 assembly had a telomere on one end. When mapping the paired-end Illumina reads generated from the 11–12 isolate and the Al-1 isolate back to the PacBio-based assemblies of their respective isolates, > 96% of the trimmed reads mapped back to the assembly, indicating that the assemblies were of high quality and that the base calling could be largely confirmed with a different sequencing technology. A lower percentage of the paired-end reads mapped from the isolate of origin to the other sequenced isolate, indicating some genomic divergence between the isolates. Overall, the assemblies had good BUSCO scores using both the eukaryotic (< 98%) and the fungal (< 99%) databases, indicating that the core set of genes were fully present and correctly assembled within each assembly (Table 1).

Transposable elements (TEs) were identified in each genome using RepeatMasker (Table 2). Overall, the total number of transposable elements identified in each genome assembly was similar, with 2882 TEs identified in the 11–12 assembly and 2703 TEs in the Al-1 assembly. Major types of TEs identified included Class I retrotransposons, including LTRs (long terminal repeats) and LINES (long interspersed nuclear elements) as well as Class II transposons including DNA transposons and helitrons [22]. Some groups of transposons were unique to the 11–12 isolate, including the L2 category of LINE transposons that were found on 8 of the 13 main contigs as well as the CRE type of LINE transposon that was only found on Contig 8. The Penelope type of LINE was only present in the 11–12 genome and is a unique type of TE that is typically associated with animal genomes [23]. Within the 11–12 genome, this TE type was more prevalent in the smaller contigs than in the 13 main contigs. Both genomes had a large number of Copia and Gypsy LTRs, which are known to be both abundant and diverse in fungal genomes [24]. In contrast, only the 11–12 genome contained endogenous retrovirus 4 (ERV4) LTRs, which were only found within the smaller contigs of the genome and have previously been identified in other fungal genomes [25].

**Table 2:** Transposable elements identified in *Macrophomina phaseolina* genomes from an isolate recovered from strawberry that represents the strawberry genotype (11–12) and an isolate recovered from alfalfa (Al-1) that represents another genotype

Transposable Element Category	11–12	Al-1
DNA	490	398
LINE/Penelope	109	
LINE/Tad1	269	179
LINE/CRE	1	
LINE/L2	12	
LINE (other)	189	158
LTR/Copia	550	385
LTR/Gypsy	1173	1522
LTR/ERV4	17	
LTR (other)	59	43
RC/Helitron	13	15

*LINE* Long interspersed nuclear element

*LTR* Long terminal repeat

*RC* Rolling circle

### Genome annotation for *M. phaseolina* isolates 11–12 and Al-1

MAKER was used to annotate the genomes of 11–12 and Al-1 using a set of protein evidence from closely related fungi and an RNA-Seq based transcript dataset from the 11–12 isolate. MAKER used several de novo gene predictors including AUGUSTUS, SNAP, and GENEMARK to begin the annotation process for these two *M. phaseolina* isolates. A final list of MAKER standard genes was created from the predicted genes that had Pfam support and or transcript evidence. In total, the 11–12 isolate contained 14,103 annotated genes and the Al-1 isolate contained 13,443. For the Al-1 genome, all of these annotated genes are on the 13 main contigs, with no MAKER-annotated genes found on the 41.5 kb contig or the four contigs representing the mitochondrial genome. Alternatively, there are 146 annotated genes on the 47 smaller contigs (4726 to 99,276 bp) of 11–12, ranging from 0 to 18 annotated genes per contig. The functional (Pfam) annotations of these genes identified on the 11–12 small contigs included helicases, retrotransposons, translation initiation factors, oxidoreductases, and aspartyl proteases.

The carbohydrate-active enzyme composition of a fungus can be used to determine its ecological niche and host specificity. To compare the carbohydrate-active enzymes from the two sequenced *M. phaseolina* isolates with other plant pathogenic and non-plant associated fungi, the carbohydrate-active enzymes encoded by each fungus in the comparison were classified using the CAZy database (Table 3). Carbohydrate-active enzymes are classified into six families, and the observed enzyme compositions are conserved within the *M. phaseolina* isolates. As expected, this comparison shows that the polysaccharide lyase (PL) gene family expanded in the plant pathogenic fungi relative to the non-plant associated fungi (e.g., *A. niger* and *N. crassa*). The PLs encoded in the *M. phaseolina* cleave different forms of pectins, such as pectate lyases in the PL1, PL3, and PL9 families, and rhamnogalacturonan lyases in the PL4 family. Additionally, the glycoside hydrolase (GH) enzymes (e.g., GH88 and GH105) that degrade the products generated by PL enzymes are also found in the *M. phaseolina* genomes. The enhanced capacity in the pectinolytic machinery suggests strong plant cell wall degradation activity that likely enables their necrotrophic infection and colonization of plant tissue.

**Table 3:** Carbohydrate-Active Enzymes of *Macrophomina phaseolina* isolates, 11–12, Al-1, and jute, and other fungi including *Aspergillus niger, Neurospora crassa, Fusarium vertcilliodes, and Verticillium dahliae*

CAZY families	Fungal Species						
	*M. phaseolina*	*M. phaseolina*	*M. phaseolina*	*A. niger*	*N. crassa*	*F. verticillioides*	*V. dahliae*
	Fungal Isolate Names						
	11–12	Al-1	MPI-SDFR	ATCC 1015	OR74A	7600 v2	VdLs.17
AA^a^	170	161	172	113	75	145	104
CBM^b^	23	24	26	29	22	43	31
CE^c^	120	117	124	91	46	140	77
GH^e^	327	335	336	310	222	414	292
GH88	1	1	1	1	0	2	3
GH115	2	2	2	0	1	2	4
GT^d^	128	127	126	148	116	190	120
PL^f^	26	26	29	11	4	24	35
PL1	9	8	9	8	1	11	16
PL3	10	11	11	0	1	7	11
PL4	5	5	5	2	1	3	4
PL9	1	1	1	0	0	2	2

^a^*AA* Auxiliary activities

^b^*CBM* carbohydrate-binding modules

^c^*CE* Carbohydrate esterase

^d^*GT* glycosyltransferases

^e^*GH* glycoside hydrolases

^f^*PL* polysaccharide lyases

### Genome sequence comparison between 11-12 and Al-1 reveals large-scale genome rearrangement

**Fig. 1:**
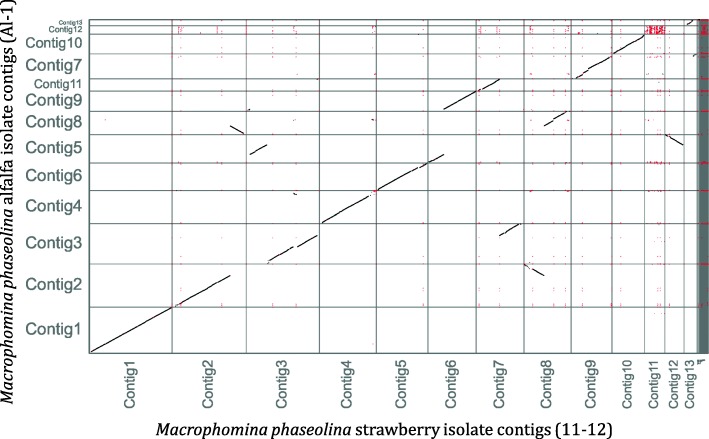
MUMmer-based Assemblytics dot plot comparing the genomes of *Macrophomina phaseolina* isolates 11–12 (strawberry genotype) and Al-1 (non-strawberry genotype)

When comparing the full genome assembly of 11–12 to Al-1, large scale structural rearrangements and smaller scale indels and SNPs were observed. The dot plot from a MUMmer analysis visualized using Assemblytics indicates that the genomes are largely collinear (Fig. 1). Some contigs have been inverted or translocated. For example, a portion of Contig 2 of the 11–12 assembly is collinear and found inverted and translocated to Contig 8 of the Al-1 assembly. Large portions of 11–12 Contigs 3, 6, and 7 are also shown to be collinear and translocated to a different contig of the Al-1 isolate. Of the larger contigs, Contig 8 shows the most changes with large portions broken and or translocated and inverted in the Al-1 assembly relative to the 11–12 assembly.

Similar pairwise whole-genome comparisons were made between the 11–12 assembly and the Illumina assemblies of other *M. phaseolina* isolates using MUMmer and Assemblytics. Overall, the genomes of all isolates, either pathogenic on strawberry or nonpathogenic on this host, were collinear with the 11–12 strawberry isolate, with some small segments of the genome showing translocations or inversions but with none of these rearrangements being consistently associated with the strawberry genotype. In general, the genomes of isolates that were nonpathogenic on strawberry exhibited more insertions and deletions compared to the 11–12 assembly, with a higher proportion of deletions, compared to those identified among the isolates pathogenic on strawberry. However, a couple of pathogenic isolates also had a high number of insertions and deletions compared to the 11–12 assembly and some nonpathogenic isolates had very few indels, indicating that using the trends of indels alone is not an accurate predictor of pathogenicity on strawberry. The different sequencing technologies used for the genome assemblies may affect the analyses given that the 11–12 long-read assembly was composed largely of 13 contigs while the Illumina-based assemblies of the other *M. phaseolina* isolates contain over 1000 contigs each.

**Fig. 2:**
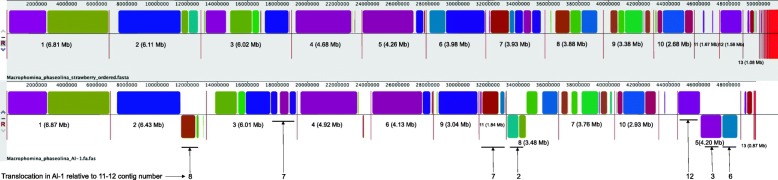
A progressive MAUVE alignment comparing the genome of the 11–12 isolate of *Macrophomina phaseolina* (top) to the Al-1 isolate genome (bottom). Numbers under the contigs identify the contig number with its size following in brackets. Translocations in isolate Al-1 relative to 11–12 are noted in the bottom of the figure

A progressive MAUVE analysis between the 11–12 and Al-1 assemblies revealed translocations and inversions as well as several syntenic blocks between the two genomes (Fig. 2). Contig 1 of both assemblies, which is over 6 Mb and the longest contig of each assembly, has two syntenic blocks. Some portions of Contig 8 of the Al-1 genome are similar to Contig 2 of the 11–12 assembly but are inverted. The third contig of each assembly has partial synteny, but a portion of the 11–12 contig can be found predominantly on Al-1 Contig 5 (inverted) and to a lesser extent Contigs 4 and 9 (Fig. 1). A portion of the Al-1 genome syntenic to Contig 6 of the 11–12 assembly is inverted and present on Contig 5 of the Al-1 assembly along with portions of 11–12 Contigs 3 and 12. Several blocks of the 11–12 Contig 7 can be found on the Al-1 Contig 3 and 11. Contig 8 of 11–12 has a region from position 359,168 to 449,477 that appears to be a unique sequence region without a clear corresponding match in the Al-1 assembly. Contig 11 of the 11–12 assembly also seems to contain unique genomic regions and does not have many syntenic regions with Al-1.

### Identification of gene clusters from *M. phaseolina* unique to the main strawberry genotype

**Fig. 3:**
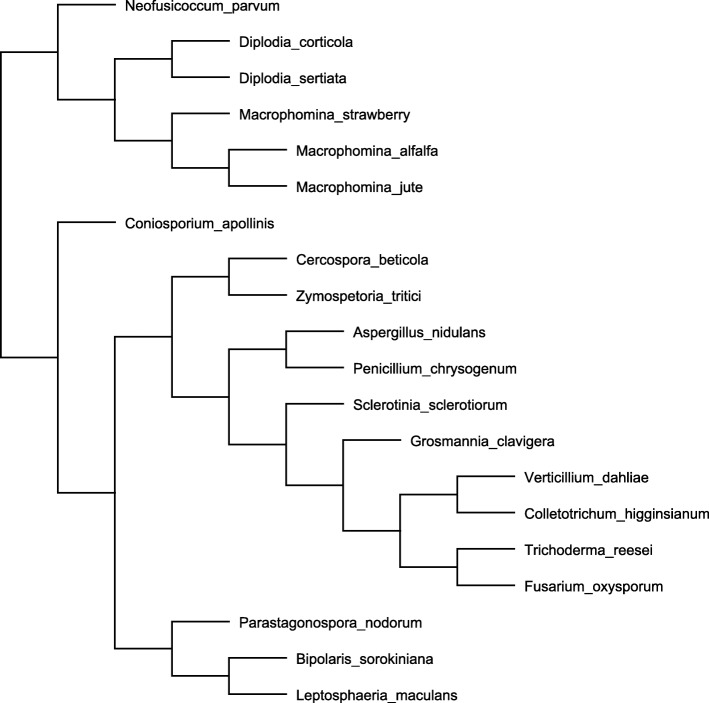
Phylogenetic tree of three *Macrophomina phaseolina* isolates and closely related fungal species using OrthoFinder

An analysis of all the orthologous genes, including the jute *M. phaseolina* assembly [1] as well as seventeen other taxa, including several other fungi within the Dothideomycetes class (Additional file 2: Table S2), were analyzed with OrthoFinder. In the resulting phylogenetic tree, the isolates of *M. phaseolina* were tightly grouped with each other and with the other members of the Botryosphaeriales order, as expected (Fig. 3; Percentage of shared orthologous genes between species is in Additional file 3: Table S3). This phylogeny also indicated isolates of *M. phaseolina* from alfalfa and jute were more closely related to each other than to the strawberry isolate, providing additional support that the host specificity aspect of the strawberry isolate genotype may be associated with evolutionary divergence. This phylogeny helped to inform the selection of isolates that were used to create OrthoVenn2 comparisons with smaller groupings of isolates. The OrthoFinder analysis also identified 2 unique orthogroups for the main strawberry genotype of *M. phaseolina* with 6 genes represented. The isolate from alfalfa did not have any specific orthogroups and the isolate from jute had 4 specific orthogroups.

**Fig. 4:**
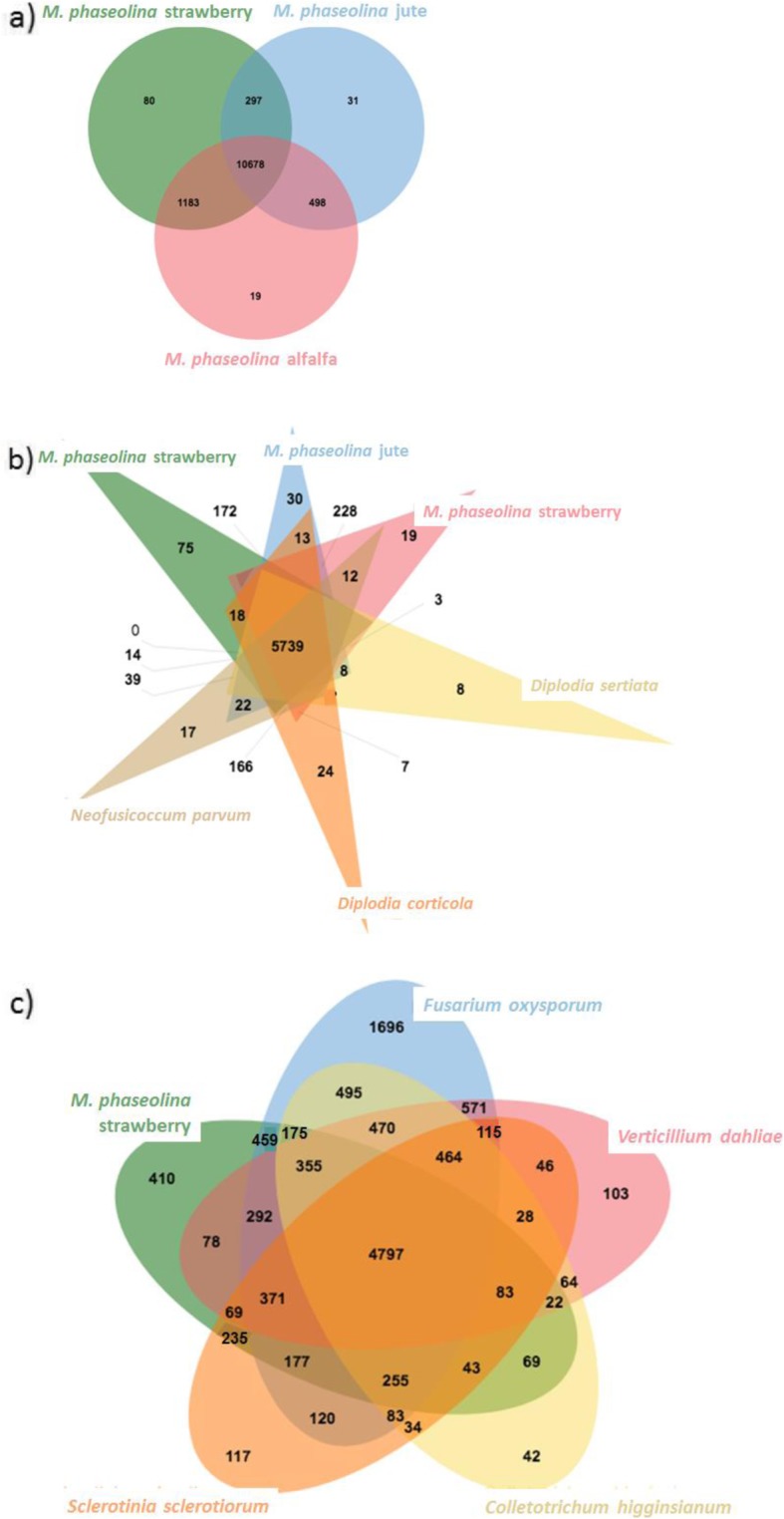
OrthoVenn diagram comparisons of *Macrophomina phaseolina* isolate 11–12 to **a**) two other *M. phaseolina* isolates from jute and alfalfa (Al-1). **b** other Botryosphaeriales fungi and **c**) other fungi that commonly infect strawberry

In an analysis with OrthoVenn2 using the *M. phaseolina* protein sequences from strawberry, jute, and alfalfa isolates, 80 clusters containing a total of 223 proteins were found to be unique to the strawberry genotype with 1338 singletons also identified as being unique (Fig. 4a). Within the group of 80 unique clusters, enriched GO slim terms, included those associated with a molecular function (nucleotide binding, oxidoreductase activity, hydrolase activity, and cofactor binding) and those associated with biological process (pyrimidine nucleotide biosynthetic process, cellular aromatic compound metabolic process, and others). Common among all three *M. phaseolina* isolates were 10,678 orthologous clusters with enriched GO terms associated with oxidoreductase activity (115 groups, *p*-value 2 × 10^− 6^) and terpenoid biosynthetic process (48 groups, *p*-value 6.5 × 10^− 5^). These two enriched GO terms provide support and additional insight into the potential infection mechanisms of *M. phaseolina* relative to other fungi*.* Oxidoreductase activity in terms of hydrogen peroxide production has been positively correlated with virulence in some strains of *M. phaseolina* on chickpea and sunflower [26] and terpene-derived secondary metabolites produced by filamentous fungi like *Fusarium* have been implicated in toxicity to plants [27].

An analysis between the selected fungi within the Botryosphaeriales order with OrthoVenn2 showed that the *M. phaseolina* isolate from strawberry had 75 unique clusters of orthologous genes with enriched GO slim terms including phosphorus metabolic process and oxidoreductase activity (Fig. 4b). The strawberry isolate also had 1231 singletons without an orthologous gene. Within the same OrthoVenn2 analysis, the three *M. phaseolina isolates* uniquely shared 1729 orthologous groups which had enriched GO terms including the molecular function of oxidoreductase activity (80 groups, *p*-value < 1 × 10^− 9^) and the biological processes of pathogenesis (29 groups, *p*-value 5 × 10^− 8^) and terpenoid biosynthetic process (14 groups, *p*-value 3.6 × 10^− 4^). All of the fungi in this analysis shared 5739 orthologous clusters, which included enriched GO terms including the biological processes of pathogenesis (48 groups, *p*-value 9 × 10^− 10^), terpenoid biosynthetic process (19 groups, *p*-value 2.4 × 10^− 9^), and vesicle-mediated transport (44 groups, *p*-value 2.8 × 10^− 7^). Several GO terms related to oxidoreductase activities were also enriched among all of the fungi in this analysis.

Another OrthoVenn2 analysis among genera of fungi that commonly infect strawberry – *Macrophomina, Fusarium, Colletotrichum, Verticilium*, and *Sclerotinia* – had 410 unique orthologous groups identified in the *M. phaseolina* isolate from strawberry along with 4110 singletons (Fig. 4c). Enriched GO terms for the unique orthologous groups include the molecular function of oxidoreductase activity (15 groups, *p*-value 1.3 × 10^− 12^) and the biological processes of shamixanthone biosynthetic process (3 groups, *p*-value 2.5 × 10^− 4^) and terpenoid biosynthetic process (5 groups, *p*-value 2.5 × 10^− 4^). All five fungi shared enriched GO terms including those associated with the biological process of transmembrane transport (60 groups, *p*-value 1.5 × 10^− 14^), the molecular function of zinc ion binding (18 groups, p-value 1.1 × 10^− 13^), and the biological process of pathogenesis (31 groups, *p*-value 8.4 × 10^− 10^). Also enriched were genes with GO terms that are associated with the breakdown of cell walls and thus perhaps pathogenicity, including polysaccharide catabolic process (14 groups, *p*-value 8.3 × 10^− 8^), cellulose catabolic process (11 groups, *p*-value 1.5 × 10^− 6^), and xylan catabolic process (7 groups, *p*-value 1 × 10^− 5^).

### Identification of candidate *M. phaseolina* genes associated with isolates pathogenic on strawberry

A comparative genomics approach using the annotated genome from the strawberry genotype was used to identify candidate genes that may be unique to *M. phaseolina* isolates that are capable of infecting strawberry. The sequencing reads from each of the 30 other sequenced isolates of *M. phaseolina* – including isolates that were both pathogenic and not pathogenic on strawberry - were perfectly mapped to the 11–12 genome (Table 4). These 30 isolates were selected because they represented the breadth of *M. phaseolina* genetic diversity and were used in a companion project to find a genomic locus that was used as a diagnostic tool to detect isolates pathogenic on strawberry [28]. In order to focus on differences only within the annotated genes in this study, HT-Seq was used to count the number of reads that mapped to each annotated gene model. Genes with no or low counts in the isolates that were not pathogenic on strawberry and with high counts in the isolates that were considered pathogenic on strawberry were selected after the first round of read mapping. Within CLC Workbench, BLAST was used to compare the gene model from the 11–12 isolate to similar genes in the other isolates to identify potential truncations or SNPs that could eliminate a candidate if these mutations were to cause significant changes in the predicted protein sequence among isolates in the main strawberry genotype. Non-synonymous mutations in candidate genes among the isolates non-pathogenic on strawberry allowed some candidate genes that had a high mapped read count to a candidate gene model in 11–12 to remain as pathogenicity-associated gene candidates. After this selection process, 10 candidate genes (Table 5) were identified, and all of them are located within a ~ 200 kb region of Contig 8 of the 11–12 assembly. Interestingly, this region is broken in a dotplot alignment between 11-12 and Al-1 (Fig. 1) and shows rearrangements and indels within the MAUVE alignment (Fig. 2).

**Table 4:** All isolates of *Macrophomina phaseolina* in addition to isolates 11–12 and Al-1 that were sequenced with Illumina and pathogenicity tested

Isolate	Host of isolate origin	Location of isolate origin (California)	Pathogenic on Strawberry	NCBI Data Accession
11–21	Cantaloupe	Los Banos	No	SAMN09764508
11–22	Cantaloupe	Los Banos	No	SAMN09764509
13–10	Watermelon	Imperial County	No	SAMN09764510
13–11	Watermelon	Imperial County	No	SAMN09764511
14–177	Sunflower	Imperial County	No	SAMN09764512
14–24	Strawberry	Stanislaus County	No	SAMN09764513
14–26	Strawberry	Ventura County	No	SAMN09764514
14–27	Strawberry	Ventura County	No	SAMN09764515
14–4	Almond	Fresno	No	SAMN09764516
14–45	Lima bean	Santa Clara County	No	SAMN09764517
14–48	Lima bean	Santa Clara County	No	SAMN09764518
16–13	Strawberry	Santa Barbara County	No	SAMN09764519
11–14	Strawberry	Ventura County	Yes	SAMN09764520
11–5	Strawberry	California nursery	Yes	SAMN09764521
12–27	Strawberry	Santa Cruz County	Yes	SAMN09764522
13–30	Strawberry	Monterey County	Yes	SAMN09764523
13–45	Strawberry	Monterey County	Yes	SAMN09764524
14–134	Strawberry	Santa Clara County	Yes	SAMN09764525
14–140	Pepper	Santa Clara County	Yes	SAMN09764526
14–170	Strawberry	Oxnard County	Yes	SAMN09764527
14–20	Strawberry	Ventura County	Yes	SAMN09764528
14–21	Strawberry	Ventura County	Yes	SAMN09764529
14–22	Strawberry	Stanislaus County	Yes	SAMN09764530
14–62	Strawberry	Santa Maria County	Yes	SAMN09764531
15–47	Strawberry	Salinas	Yes	SAMN09764532
15–69	Strawberry	Salinas	Yes	SAMN09764533
15–87	Strawberry	California nursery	Yes	SAMN09764534
15–89	Strawberry	California nursery	Yes	SAMN09764535
16–14	Strawberry	San Luis Obispo	Yes	SAMN09764536
16–15	Strawberry	San Luis Obispo	Yes	SAMN09764537

**Table 5:** Candidate genes associated with isolates in the main strawberry genotype that are aggressive on strawberry

Candidate Gene	Start	Stop	Gene Length	Pfam Domain	Signal Peptide
M11_12_v1_01446	467,679	468,583	904	Ribosomal protein L36	no
M11_12_v1_01448	458,879	462,158	3279	Myb-like DNA-binding domain	no
M11_12_v1_01455	468,323	470,605	2282	NA	no
M11_12_v1_01458	523,857	525,833	1976	Major Facilitator Superfamily, Sugar (and other) transporter, Organic Anion Transporter Polypeptide (OATP) family	no
M11_12_v1_01460	530,438	531,136	698	Fungal Zn(2)-Cys(6) binuclear cluster domain, Zinc knuckle	no
M11_12_v1_01463	516,694	519,283	2589	Aldehyde dehydrogenase family	no
M11_12_v1_01466	519,472	521,373	1901	Cytochrome P450	no
M11_12_v1_01490	654,497	656,140	1643	NA	no
M11_12_v1_01493	623,466	624,632	1166	Membrane dipeptidase (Peptidase family M19)	yes
M11_12_v1_01501	655,801	656,697	896	Protein of unknown function (DUF3408)	no

The candidate genes identified in Table 5 were typically absent in all 13 of the nonpathogenic isolates and present in all 19 of the isolates of the strawberry genotype pathogenic on this host except in the case of isolates 14–26 and 14–27. These isolates were recovered from diseased strawberry plants in Ventura, California, are not in the strawberry genotype, and were found to be nonpathogenic on strawberry in the toothpick inoculations, yet had sequences of candidate genes that were identical to those found in 11–12. Several of these genes, including M11_12_v1_01446, M11_12_v1_01448, M11_12_v1_01460, M11_12_v1_01490, M11_12_v1_01493, and M11_12_v1_01501 were also listed as a member of a unique orthologous grouping or as singletons for *M. phaseolina* of strawberry in all the OrthoVenn2 comparisons. Additionally, for some candidate genes – M11_12_v1_01458, M11_12_v1_01460, M11_12_v1_01463, and M11_12_v1_01466 - isolates nonpathogenic on strawberry, Al-1 and 14–4, had similar gene sequences, but SNPs or indels changed the predicted protein sequence when compared to that of 11–12. For gene M11_12_v1_01493, the predicted proteins from all the nonpathogenic isolates except for 14–26 and 14–27 were truncated compared to the 11–12 gene model and were predicted to be metalloproteinases while the full-length gene model from 11-12 was predicted to encode a membrane dipeptidase. This is also the only gene among the candidate genes that was predicted by SignalP to have a signal peptide. When looking at the transcript support for each candidate gene model, all the genes except for M11–12_v1_01448, M11_12_v1_01493, and M11–12_v1_01501 had full or partial support from the transcripts predicted by Trinity using the 11–12 RNA-Seq datasets provided during the gene annotation. Furthermore, when examined individually, many of the RNA-Seq datasets provided support for the expression of the candidate genes, with M11_12_v1_01446, M11_12_v1_01448, M11_12_v1_01463, and M11_12_v1_01501 being highly expressed in all the transcript datasets. A couple of genes, M11_12_v1_01455 and M11_12_v1_01493, had higher gene expression in the dataset from the crown tissue medium compared to other growth conditions, but additional experiments are needed to confirm the significance of this observation.

## Discussion

Overall, this study provides two high-quality assemblies for *M. phaseolina* and explores genetic differences between *M. phaseolina* isolates collected from different hosts. The use of third generation sequencing technology, including long read PacBio sequencing, allowed for a genome that was more complete and contiguous compared to the previously published genome that was assembled using older short-read technology [1]. Furthermore, the assemblies presented in this manuscript are of very high quality, with thorough genome polishing completed using PILON with Illumina-based mate pair and paired-end libraries. The improved sequencing technology was used at a much higher coverage (150x for PacBio and 200x for Illumina) compared to the 66x coverage for the jute assembly. Beyond using advancements in sequencing technology, this work also confirmed the accuracy of assemblies with new methods in genome scaffolding from Dovetail Genomics, which used Hi-C proximity ligation, the HiRise software, and PacBio gapfilling. This resulted in a final assembly with no gaps within the contigs in the case of the Al-1 genome and 13 gaps in the case of the 11–12 genome. The total genome size for all three isolates was similar, with the strawberry isolate at 51.3 Mb, the alfalfa isolate at 49.8 Mb, and the jute isolate at 48.9 Mb. Compared to the 454 and Illumina short read technology used in the assembly of the jute isolate [1], the use of the long-read PacBio technology and Dovetail scaffolding of Hi-C data greatly decreased the total number of scaffolds, improved the L75, and increased the length of the longest contig, as shown in Table 1. Overall, this approach has produced very high-quality genomes with an N50 of 4.3 and 5.0 Mb for the strawberry and alfalfa isolates, respectively. This is a significant improvement over the previous reference assembly of *M. phaseolina* of an isolate recovered from jute, which had an N50 of 151 kb [1].

While the assemblies presented in this work are very high quality and are an improvement over the published genome from a jute isolate, there are areas where future work could improve the quality of the strawberry genotype assembly to make a fully complete chromosome-level assembly. First, one shortcoming of this study is that FALCON, an assembler designed for diploids, was used to assemble a haploid genome [29]. In this case, the primary contig file was used as the basis for the final assembly, but a smaller associate contig file was also produced by the assembler which contained ~ 50 small contigs per assembly that were each < 70 kb. These contigs were compared by BLAST to the final assembly and were found to be fully contained within the primary contigs and were discarded. Similarly, several shorter contigs were identified in the primary contig-based assembly of both isolates even following the Dovetail scaffolding, and many of these contigs were fully redundant within the genome. Because the sequences were fully duplicated within larger chromosomes with nearly 100% sequence identity, these shorter contigs were considered assembly errors – perhaps due to the exceedingly high amount of raw input data at nearly 200x genome coverage - and were removed to produce the final number of contigs in the final assembly.

Still, there are some smaller contigs, especially within the 11–12 assembly, with a largely unknown identity or placement within the larger genome. A couple of these contigs that are less than 100 kb are the mitochondrial genome of *M. phaseolina*, including Contig 15 in the 11–12 assembly and Contigs 14, 16, 17, and 18 in the Al-1 assembly. In the Al-1 assembly, only Contig 15, a 41,517 bp contig, remains unplaced in the final assembly and does not contain any annotated transcripts based on annotation with MAKER. In contrast, the 11–12 assembly has 46 small contigs representing the nuclear genome remaining in the assembly, with 34 of them having at least one annotated gene and repeat. Unlike the smaller contigs of Al-1, the small contigs of the 11–12 genome had a greater number and diversity of transposable elements, including unique Penelope LINE transposons and ERV4 LTR transposons which may contribute to the difficulty of their scaffolding compared to Al-1. Chromosomal sized bands in pulsed field gel electrophoresis were observed that corresponded to the 0.87 and 1.24 Mb contigs of the Al-1 assembly (smaller bands were not seen); while a band corresponding to the 1.07 Mb contig of the 11–12 assembly was observed, a corresponding scaffold for a 0.79 Mb band in the gel was not (Additional file 4: Figure S1). It is likely a number of the smaller contigs of 11–12 would be scaffolded to this 0.79 Mb band. Additional methods of scaffolding, including the electronic mapping used by Nabsys (Providence, RI), could help to place some of these small contigs into larger scaffolds [30]. In *Fusarium oxysporum* it has been demonstrated the smaller chromosomes rich in repeats and transposons encode genes associated with pathogenesis [15]; it is unknown if is the case with the smaller contigs of the 11–12 isolate of the strawberry genotype which also displayed high numbers of transposons on the smaller contigs.

The functional annotation of these genomes was completed using RNA-Seq data derived from the 11–12 isolate grown in 10 different conditions to ensure that variation in gene expression was captured to obtain high-quality data for annotation. This extensive set of transcriptome data was used along with a representative set of protein sequences from taxonomically related fungi to create an updated and thorough functional annotation of the *M phaseolina* genome. Despite using the most up-to-date available data and state-of-the-art computational tools to identify orthologous genes and predict gene function, functional annotations are still imperfect in that they use tools that base annotations upon proteins and domains that have been previously characterized in model organisms that are at best closely related to *M. phaseolina* and many times have only been fully characterized in different biological kingdoms. As a result, many predicted proteins are identified as a “gene of unknown function” and several of the clusters of orthologous genes determined through OrthoFinder or OrthoVenn2 don’t have predicted functions or GO annotations. These tools are still useful in sorting the data that we have based on the annotation databases that are currently available, but more work needs to be done in the field of fungal functional genomics before these predictions can be more accurate and analysis like GO term enrichment can be more biologically meaningful.

When searching for genes associated with isolates representing the strawberry genotype, 10 genes from the 11–12 assembly were identified including enzymes, proteins involved in DNA binding, and several genes with unknown function. Despite a very rigorous pipeline to identify genes that were unique to isolates in the strawberry genotype, candidate genes were also present in isolates 14–26 and 14–27 that were initially recovered from strawberry but were not pathogenic in laboratory inoculations. Since a single, highly susceptible strawberry cultivar was evaluated in determining *M. phaseolina* pathogenicity; it is possible that these isolates may be pathogenic on other strawberry cultivars. Furthermore, these isolates were part of the less than 2% of the isolates recovered from strawberry that were not in the strawberry genotype. Testing with soil-based inoculum derived from an isolate within this small subgroup revealed it had a much lower level of virulence on strawberry when compared to isolates from the strawberry clade (B. Smith and F. Martin, unpublished).

Given these considerations, the list of candidate genes generated from an extensive presence-absence analysis of genes annotated in the strawberry isolate provides a strong foundation to evaluate potential causes of *M. phaseolina* pathogencity and virulence on a strawberry host. In some cases, genes with similar Pfam annotations have been shown to have significant roles in pathogenicity and host interactions. For example, genes M_11_12_v1_01448 and M11_12_v1_01460 are both likely transcription factors with Pfam domains including the Myb-like DNA binding domain and the Zn(2)-Cys(6) binuclear cluster domain, respectively. Previous research has shown that transcription factors with the Myb-like DNA binding domain regulate the secretome in necrotrophic fungal pathogen [31] and a transcription factor with the Zn(2)-Cys(6) binuclear cluster domain has a key role in regulating virulence in a fungus infecting wheat [32]. The cytochrome P450 gene, M11_12_v1_01466, is a member of a large group of genes that was found to be present in the *M. phaseolina* genome in high numbers and may be associated with secondary metabolites and overcoming host defenses [1]. A member of the Major Facilitator Superfamily, M11_12_v1_01458, is also part of a group of genes that was found in high numbers in the *M. phaseolina* genome [1] and has been shown to be important in mediating pathogenicity in other fungi [33]. These candidate genes, especially those with unknown functions, could have novel roles in pathogencity and virulence, but to fully test whether the candidate genes found predominantly in the strawberry genotype are responsible for conferring pathogenicity to *M. phaseolina* on strawberry, a knockout study using new technology like CRISPR would need to be done [34].

Beyond examining the presence or absence of particular genes in the genome, it is important to investigate the expression and interdependence of genes responsible for enabling an isolate of *M. phaseolina* to infect strawberry. Further studies examining gene expression or transcriptional networks during infection of strawberry by *M. phaseolina* may reveal a more complex role for some of these candidate genes. It will be interesting to see if additional genes or gene expression patterns unique to the strawberry genotype are observed once the smaller contigs in the 11–12 assembly are joined into larger chromosome-sized contigs/scaffolds. The high quality genomes produced in this manuscript provides a valuable resource for future transcriptomic studies that will further elucidate which genes and pathways are involved in conferring host specificity to isolates of *M. phaseolina* infecting strawberry.

## Conclusions

In summary, this work provides high-quality annotated genome assemblies for *Macrophomina phaseolina* isolates from strawberry and alfalfa hosts. These PacBio-based assemblies are an improvement over the previously published assembly for an isolate recovered from jute in that these assemblies have fewer contigs, longer contigs, and all the raw data from the sequencing and annotation is publicly available. Comparative genomics of these more complete assemblies has revealed large-scale structural rearrangements in terms of chromosomal inversions and translocations. In addition, thirty additional isolates from strawberry and other hosts were sequenced with Illumina in this study, all of which provide valuable comparative genomics resources for this and other studies involving *M. phaseolina*. In this study, an analysis of gene presence/absence created a short list of candidate genes associated with isolates pathogenic on strawberry that may be involved in host preference to strawberry.

## Methods

### *M. phaseolina* isolate growth and DNA extraction

Cultures of *M. phaseolina* were recovered from infected plants collected in California. The 11–12 isolate was collected from strawberry in Santa Barbara County, CA, and the Al-1 isolate was collected from alfalfa in Imperial County, CA. Tissue for DNA extraction was grown on potato dextrose broth (PDB) at 28 °C for two days. The cultures were then blended with sterile water and grown for another 2 days at 28 °C to produce a hyphal mat. This tissue was strained, rinsed with sterile water, blotted dry, and flash frozen. The tissue was lyophilized and DNA was extracted using the Blood and Cell Culture kit (Qiagen, Redwood City, CA) with the modifications described in the Qiagen user-developed protocol for the isolation of genomic DNA from plants and filamentous fungi using the Qiagen Genomic tip. The only exception was that buffer G2 in the kit was used as the lysis buffer. The quality of the DNA was tested by running the samples on a 0.8% agarose gel and the concentration measured using a Qubit (Thermo Fisher Scientific, Waltham, MA). The samples were submitted for PacBio RSII sequencing to the DNA Technologies Core at the Genome Center of the University of California (Davis, CA). Fourteen SMRT cells were sequenced for the isolate representing the main strawberry genotype (11–12; SAMN08294525) and 10 SMRT cells were sequenced for an isolate recovered from alfalfa representing another genotype (Al-1; SAMN08448485). The DNA was also submitted to Michigan State University Research Technology Support Facility Genomics Core (East Lansing, MI) for mate pair library preparation and Illumina paired-end sequencing. The mate pair libraries were prepared using the Illumina Nextera Mate Pair Library Preparation Kit. The inserts for the mate pair libraries were prepared by fragmenting the DNA sample and running it on a polyacrylamide gel to separate the fragment sizes. The samples were run on an Agilent Bioanalyzer DNA 12000 chip which showed that the mate pair insert fragment ranges for isolate 11–12 were 3.5, 5.0, and 7.0 kb for expected 3, 6, and 9 kb insert libraries, respectively. These libraries were sequenced using Illumina HiSeq 2500 High Output flow cell (v4) using 125 bp paired-end sequencing with the HiSeq SBS reagents. For the Al-1 sample, mate pair inserts of 3.1, 5.7, and 8.5 kb were created by fragmentation and were sequenced on an Illumina MiSeq v2 flow cell to provide 150 bp paired-end data. DNA for these two isolates was also sent to the USDA-ARS Genomics and Bioinformatics Research Unit (Stoneville, MS) for a single MiSeq run using the same library preparation techniques noted above to provide 300 bp paired-end data. The paired-end and mate pair data for 11–12 and Al-1 is available on NCBI under accession number SAMN08294525 and SAMN08448485, respectively.

Additional sequencing was completed using DNA extracted from lyophilized tissue of 30 isolates of *M. phaseolina* using the Qiagen DNeasy Plant Mini Kit following the manufacturer’s directions for lyophilized plant tissue except the tissue lysis step was increased to one hour. This DNA was submitted to either Michigan State University Research Technology Support Facility Genomics Core or DNA Technologies Core at the Genome Center of the University of California for 150 bp paired-end sequencing using the Illumina HiSeq4000. The data can be located at NCBI under accession numbers SAMN09764508-SAMN09764537 (for a full list of isolates and the corresponding accession numbers see Table 4).

### *M. phaseolina* isolate growth and RNA extraction

Isolate 11–12 of *M. phaseolina* was grown under a variety of conditions to express a wide range of genes for RNA sequencing to be used for genome annotation. All the samples were started on PDB and were grown at 28 °C for two days. Three samples were blended with water and were transferred to 15 °C, 35 °C, or 24-h light and were grown for an additional two days. One sample was grown at 28 °C for 7 days after it was blended. Other cultures that were started on PDB were transferred to a different media by removing the growing culture with a rubber policeman and rinsing it briefly with distilled water before blending it with a new media and transferring it to a new set of petri plates to grow for 2 days at 28 °C. The other media used included PDB with 2 and 4% NaCl and PDB adjusted to a pH of 3.5 or 8 with HCl or NaOH. Additional media included 100 mM ammonium sulfate and sterilized macerated strawberry crown extract. All the fungal tissue was collected from the various growth conditions by straining the media from the tissue, rinsing the tissue with sterile water several times, and blotting the tissue dry. The tissue mat was then rolled into a cylinder and flash-frozen in liquid nitrogen and stored at − 80 °C until the RNA was extracted.

RNA was extracted using the Qiagen RNeasy Plant Mini Kit from flash-frozen tissue grown in each of the conditions described above. The RNA was submitted to the Michigan State University Research Technology Support Facility Genomics Core and was prepared using the Illumina TruSeq Stranded mRNA Library prep kit and was sequenced using 125 bp paired-end reads from the Illumina HiSeq 2500. The reads were submitted to NCBI (SAMN09699088, SAMN09699089, SAMN09699090, SAMN09699091, SAMN09699092, SAMN09699093, SAMN09699094, SAMN09699095, SAMN09699096, SAMN09699097).

### Genome assembly with FALCON

The genomes of *M. phaseolina* isolates 11–12 and Al-1 were assembled from the PacBio data using the FALCON assembly pipeline v. 0.3.0 [29]. First, the reads were converted from the raw bax.h5 format to subreads.fasta using the pbh5tools v. 0.8.0. FALCON was run for isolate 11–12 using the following parameter options: input_type = raw, length_cutoff = 10,000, length_cutoff_pre = 10,000, pa_HPCdaligner_option = −v -B128 -t16 -e.70 -l1000 -s400 -T4, ovlp_HPCdaligner_option = −v -B128 -t32 -h60 -e.96 -l500 -s1000 -T4, pa_DBsplit_option and ovlp_DBsplit_option = −× 500 -s100, falcon_sense_option = −-output_multi --min_idt 0.70 --min_cov 4 --max_n_read 200 --n_core 6, and overlap_filtering_setting = −-max_diff 300 --max_cov 500 --min_cov 5 --bestn 10 --n_core 15. FALCON was run for isolate Al-1 using the same parameters except for the following changes: length_cutoff = 15,000, length_cutoff_pr = 11,000, and overlap_filtering_setting = −-max_diff 100 --max_cov 450 --min_cov 10 --bestn 10 --n_core 15. The resulting p_ctg.fa output file was used as the input genome assembly for subsequent analyses.

### Genome assembly improvement with PILON

The mate pair and paired-end reads used to polish the FALCON-based assemblies were first trimmed using NxTrim v. 0.4.1 [35] using the –justmp and –separate options and or Trimmomatic v. 0.36 [36]. Bowtie2 (v 2.2.6) was used to map the trimmed mate pair and paired-end reads to the FALCON-based assemblies of each genome [37]. Bowtie2 was run for isolate 11–12 data using the –very-sensitive option and with the -I parameter set at 2000, 3500, or 4500 for a minimum fragment length and the -X parameter set at 5000, 6500, or 9000 for a maximum fragment length for the ~ 3.4 kb, 6 kb, and 9 kb mate pair insert libraries, respectively. Bowtie2 was run using the –very-sensitive option with the -I parameter set at 1500, 4200, or 6500 and the -X parameter set at 4500, 7200, or 10,500, respectively for the mate pair libraries that were approximately 3 kb, 6 kb, and 9 kb for isolate Al-1 data. The mapped reads were converted to sorted bam files using SAMTools v. 1.17 [38, 39] and were used as –jumps (mate-pair) or –frags (paired-end) input data for PILON v. 1.16 [19] to polish the FALCON-based assemblies. The corrected fasta file from PILON that had SNPs, indels, gaps, and local misassemblies fixed was used as the reference sequence for a second round of paired-end and mate-pair read mapping using the parameters for Bowtie2 described above. The sorted bam files from the second round of Bowtie2 mapping were used for a second PILON run using the same parameters described above. The final assembly from each second round of PILON-based polishing was used as the input assembly for Hi-C analysis by Dovetail (Santa Cruz, CA).

### Hi-C scaffolding

Hyphal mats of *M. phaseolina* from isolates 11–12 and Al-1 were collected during the log phase of growth in PDB, rinsed, and partially dried on filter paper by pulling a low vacuum. The tissue was flash-frozen in liquid nitrogen and sent to Dovetail Genomics (Santa Cruz, CA). The assembly was scaffolded following Hi-C analysis using the HiRise method and the gaps in the scaffolds were filled in with PBJelly using the previously described PacBio subreads. The contigs from the Dovetail gap-filled assembly were analyzed using CLC Genomics Workbench v. 9.5.3. The Align Contigs tool in the Genome Finishing Module was used to align the contigs to themselves using BLAST [40] with a word size of 20, a maximum e-value of 0.01, and minimum match size of 100. Contigs were eliminated from the final assembly that were more than 99.5% contained within another contig and had greater than 99.5% identity to that contig. Contigs less than 1000 bp were also removed. The contigs/scaffolds were reordered from longest to shortest and renamed so that the longest contig/scaffold was named Contig 1 and the shortest contig/scaffold was Contig n, where n equals the number of total contigs/scaffolds in the assembly. The final assemblies for isolates 11–12 (PRJNA428521) and Al-1 (PRJNA432410) are available on NCBI. Assemblies were evaluated for quality and completeness using QUAST v. 4.3 [41] and BUSCO2 v. 2.0 [42] with both the eukaryotic and fungal dataset.

### Genome annotation

MAKER [43] was used to structurally annotate both genomes, using RNA-Seq reads produced from 11-12 and predicted protein sequences from the NCBI GenBank assemblies of closely-related fungal species (*Fusarium oxysporum* GCA_00149955, *Macrophomina phaseolina* MS6 GCA_000302655, *Penicillium chrysogenum* GCA_000710275, *Grosmannia clavigera* GCA_000143105, *Aspergillus nidulans* GCA_000149205, *Trichoderma reesi* GCA_000167675, and *Podospora anserina* GCA_000226545). Trimmomatic v. 0.36 was used to trim the RNA-Seq reads using the default settings with the minimum read length set at 50. The trimmed paired reads were then mapped to each genome using STAR aligner v. 2.3.0e [44]. SAMTools v.0.1.19 was used to convert the aligned sam file into a sorted bam file. Trinity v. 2.4.0 [45] was used to create gene models using the –genome-guided_bam option, a maximum intron length of 5000, the jaccard clipping option, and the SS_lib_type RF. The gene models produced by Trinity from each RNA-Seq data set were used as EST evidence when running MAKER v. r1228. Repeat masking was done within MAKER RepeatMasker [22] using the RepBase [46] for all model organisms with soft masking. The specific transposable elements were curated from the output of RepeatMasker for each genome. De novo gene predictions were made using SNAP [47], Augustus [48] and Genemark v. 4.32 [49]. Specific MAKER options were set so that the AED_threshold was 1, the pred_flank was 200, the split_hit was 10,000, and the single_length was 250. A MAKER standard gene set was made which included genes that had evidence and or Pfam support with a cutoff of 1e-10 and encoded a protein of at least 50 amino acids. The transcripts file, protein sequences, gff files, and final assemblies are available at Data Dryad.

### Functional annotation

The MAKER standard gene set was subject to gene functional annotation using Trinotate v3 [50]. Both protein and transcript sequences were queried against the Swiss-Prot database [51] using BLASTP and BLASTX [40], respectively. In addition, protein sequences were scanned for conserved Pfam domains in the Pfam-A database [52] using HMMER v3 [53]. Both SignalP [54] and TMHMM [55] were used to identify signal peptide and transmembrane domains in the predicted protein sequences. Outputs from BLASTX, BLASTP, HMMER, SignalP, and TMHMM were used to generate the final functional annotation report. To characterize the carbohydrate-active enzymes in the fungal genome, protein sequences were searched against the dbCAN HMMdb release v7 [56], and the identified carbohydrate enzymes were assigned to appropriate CAZy enzyme classes (GHs, GTs, PLs, CEs, CBMs, and AAs).

### Assembly and sequencing of other isolates of *M. phaseolina*

Illumina sequencing data from the additional 30 isolates of *M. phaseolina* were analyzed using CLC Workbench (Table 4). The reads were trimmed using the Illumina TruSeq adapter set with a quality trim setting at 0.05 and a minimum read length of 30 bp. These trimmed reads were assembled into draft assemblies using CLC Workbench using the de novo assembly tool with an automatic bubble size and with a word size that was manually adjusted from 25 to 50 to provide the assembly with the best N50. The assemblies were used to create BLAST databases for downstream analyses within CLC Workbench and were also exported for downstream analyses using other programs. The statistics for the jute isolate MphMS6_1.0 in Table 1 were generated by using the publicly available assembly, GCA_000302655.1, which is assembled at the contig and not the scaffold level.

### Genetic structural variation

To evaluate genome structural rearrangement, *M. phaseolina* files were prepared for Assemblytics [57] using MUMmer v. 3.23 [58] with the following settings: -l 100, −c 500. The output file from MUMmer was used as the input for Assemblytics using the unique sequence length of 10,000, a maximum variant size of 10,000, and a minimum variant size of 1. A progressive MAUVE alignment using MAUVE v. 2.3.1 [59] was also run with the final 11–12 assembly as the reference sequence to which the final Al-1 assembly was aligned.

### Functional comparative genomics

Orthologs between *M. phaseolina* from strawberry and other isolates of *M. phaseolina* as well as 17 other closely-related fungal species were identified using OrthoFinder v. 1.1.3 [60]. The fungal taxonomy and protein sequences used in this analysis can be found in Additional file 1: Table S1. OrthoVenn2 was used to create plots and to do additional GO term enrichment analyses of specific groups [61]. All enriched GO term groups had an E-value of 0.01 and an inflation value of 1.5 as specified with the OrthoVenn2 online toolkit.

### Identification of genes unique to the main strawberry genotype

Illumina-based paired-end DNA sequencing was analyzed for 12 isolates not pathogenic on strawberry and 18 isolates pathogenic on this host to identify genes that are unique to *M. phaseolina* isolates from the strawberry genotype. These reads were trimmed using Trimmomatic and were mapped to the 11–12 genome using STAR [44] with the –outFilterMultimapNmax set to 1 and the –outFilterMismatchNmax set to 0 so that only the reads that were perfectly mapped to the genome were included in the output .sam file. The file was then converted into a sorted .bam file that was used as an input for HTSeq-count from HTSeq v. 0.6.1p1 [62]. HTSeq-count was used with the -f bam, −s no and -t gene parameters using the gff3 file from 11-12 as the gene model input. The read counts per gene from each of the isolates was analyzed using the DESeq package v. 1.32.0 [63] in R v. 3.5.0. Each set of read counts were normalized according to the library size and the counts for each gene for each isolate were exported to Excel. Excel was used to sort the count data files to identify genes that had no reads mapping from the nonpathogenic isolates and had several reads mapping from the pathogenic isolates. These genes were considered as candidate genes unique to the strawberry genotype to be used for downstream analyses. Additional analysis of candidates was done using the BLAST functions in CLC Workbench in which the 11–12 candidate gene sequences were used as queries to BLAST against a local database created in CLC Workbench from the assembly of each isolate. The top BLAST hit from each assembly was considered as the homolog for the candidate gene in that isolate and was used to manually check for the presence of indels or SNPs compared to the gene model from the 11–12 isolate using sequence alignments in CLC Workbench.

### Pathogenicity testing

*M. phaseolina* isolates were tested for pathogenicity on strawberry by growing cultures on potato dextrose agar (PDA) at 25 °C and allowing the isolates to colonize sterile toothpicks placed on top of the growing cultures for 10 days. The colonized toothpicks were used as the inoculum source. Prior to inoculation, strawberry plants of cultivar (cv.) Albion were grown in the greenhouse from crowns for 28 days. Plants were inoculated by making a 6 mm deep hole in each crown with a metal probe and then placing the colonized toothpick inside the hole. Sterile toothpicks were placed in each crown as a negative control and toothpicks colonized with isolate 11–12 were used as a positive control. For each trial, 8 plants were inoculated per treatment and were placed in a growth chamber set at 30 °C and were watered as needed. The plants were rated from 1 to 4 weeks post inoculation using a 1–3 scale in which a rating of 1 was a symptomless plant, a rating of 2 indicated a moderate amount of wilt and die back, and a rating of 3 indicated complete collapse and death of the plant. The final ratings were analyzed using the nonparametric Kruskal-Wallis test in R followed by pairwise comparisons using the Wilcoxon rank sum test; isolates were considered to be pathogenic if the adjusted *P* value compared to the negative control was < 0.05 [64] (Table 4). Each isolate was tested in 1–3 separate trials.

## Supplementary information

**Additional file 1: Table S1.** List of all of the contig lengths for the 11–12 and Al-1 *Macrophomina phaseolina* genome assemblies.

**Additional file 2: Table S2.** List of fungal species used in OrthoMCL analysis.

**Additional file 3: Table S3.** Percentage of shared orthologous genes for 20 species of fungi closely related to and including three isolates of *Macrophomina phaseolina.* Data corresponds to Fig. 3.

**Additional file 4: Figure S1.** Pulsed-field electrophoresis gel of *Macrophomina phaseolina* isolates 11–12 and Al-1 run on a BioRad CHEF DR-II in a 0.8% BioRad certified megabase agarose gel with a ramping interval of 100–180 min for 65 h at 4 v/cm run at a constant 4 °C temperature. Size markers in the left lanes are *Hansenula wingei* and *Saccharomyces cerevisiae.*

## Data Availability

Digital storage of fasta and .gff files for the genome can be found at Data Dryad: 10.5061/dryad.zkh18935h. The datasets described in this manuscript are available through NCBI. Illumina sequencing of 30 isolates of *M. phaseolina* – BioProject PRJNA484400. RNA sequencing of 11–12 libraries – BioProject PRJNA428521. Sequencing and genome assembly for Al-1 isolate – BioProject PRJNA432410. Sequencing and genome assembly for 11–12 isolate – BioProject PRJNA428521.

## Abbreviations

11–12: *Macrophomina phaseolina* isolated from strawberry in 2011
Al-1: *Macrophomina phaseolina* isolated from alfalfa
BLAST: Basic Local Alignment Search Tool
BUSCO: Benchmarking Universal Single Copy Orthologs
CAZY: Carbohydrate-Active enzymes
CRISPR: Clustered Regularly Interspaced Short Palindromic Repeats
GO: Gene Ontology
NCBI: National Center for Biotechnology Information
PacBio: Pacific Biosciences
PDA: Potato dextrose agar
PDB: Potato dextrose broth
QUAST: Quality Assessment Tool for Genome Assemblies
SMRT: Single Molecule Real-Time
SNP: Single nucleotide polymorphism
