# Supplementary figures and images for: Assembly, annotation, and comparison of Macrophomina phaseolina isolates from strawberry and other hosts
Source: BMC Genomics. 2019 Nov 4;20:802. doi: 10.1186/s12864-019-6168-1 (PMC6829926; doi:10.1186/s12864-019-6168-1)

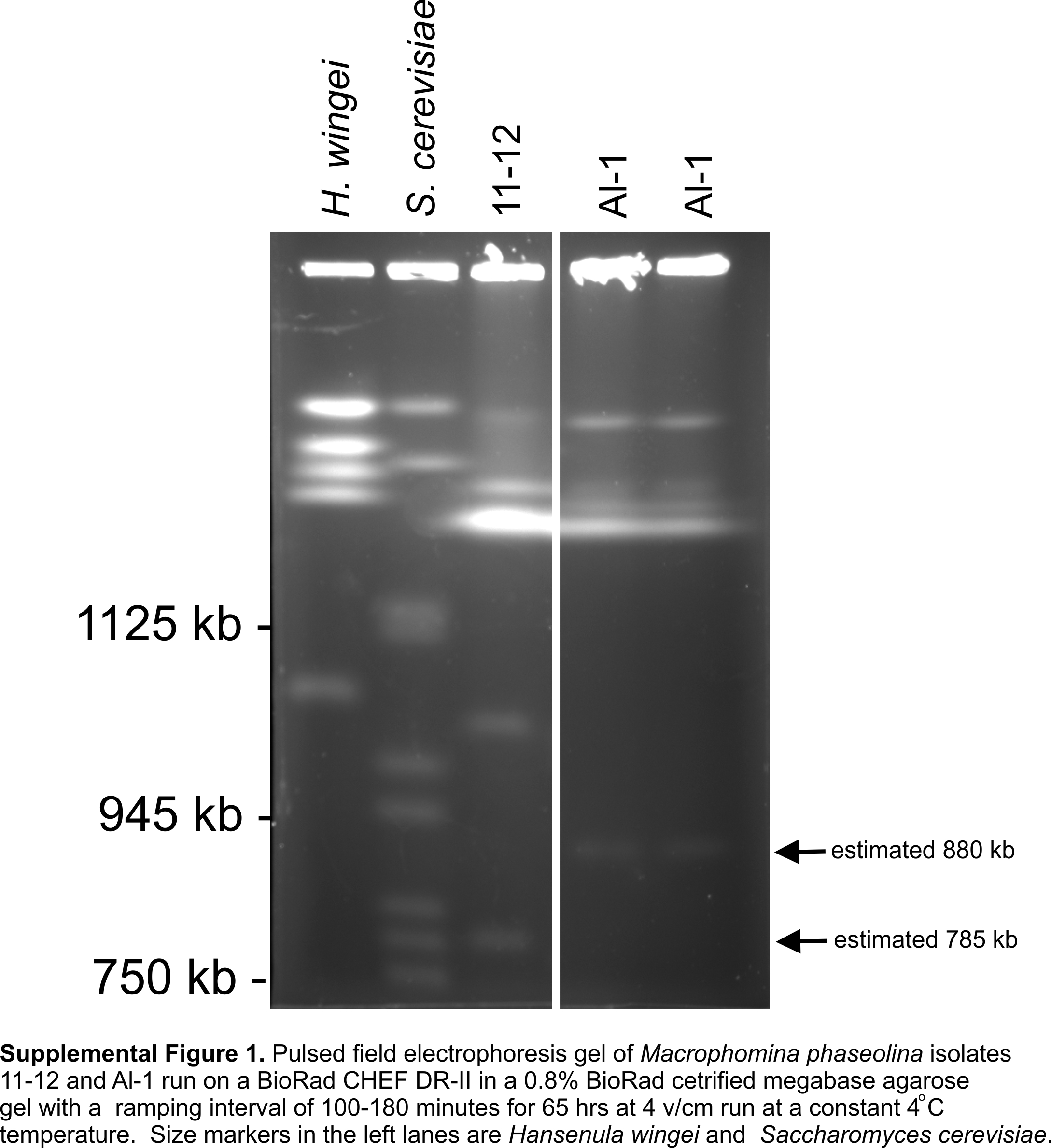

Supplement: Supplementary file 4 — Additional file 4: Figure S1. Pulsed-field electrophoresis gel of Macrophomina phaseolina isolates 11–12 and Al-1 run on a BioRad CHEF DR-II in a 0.8% BioRad certified megabase agarose gel with a ramping interval of 100–180 min for 65 h at 4 v/cm run at a constant 4 °C temperature. Size markers in the left lanes are Hansenula wingei and Saccharomyces cerevisiae. [file 12864_2019_6168_MOESM4_ESM.jpg]
